# Trends in mortality from pulmonary hypertension amongst population with congenital heart disease in the United States from 1999 to 2020: A CDC WONDER analysis

**DOI:** 10.1016/j.ijcchd.2025.100602

**Published:** 2025-06-21

**Authors:** Allahdad Khan, Tehreem Asghar, Kumail Mustafa Ali, Muhammad Saeed, Saniya Ishtiaq, Fatima Ashfaq, Jamil Nasrallah, Laiba Yumn, Usman Ali Akbar, Peter Collins, Raheel Ahmed

**Affiliations:** a Department of Medicine, Nishtar Medical University, Multan, Pakistan; b Akhtar Saeed Medical & Dental College, Lahore, Pakistan; c Department of Medicine, Jinnah Sindh Medical University, Karachi, Pakistan; d D. G. Khan Medical College, Dera Ghazi Khan, Pakistan; e Rawalpindi Medical University, Rawalpindi, Pakistan; f Department of Medicine, Faculty of Medical Sciences, Lebanese University, Beirut, Lebanon; g West Virginia University, Camden Clark Medical Center, West Virginia, United States; h National Heart and Lung Institute, Imperial College London, London, United Kingdom

**Keywords:** Congenital heart disease, Pulmonary hypertension, Mortality, CDC WONDER

## Abstract

**Background:**

Congenital heart disease (CHD) is an umbrella term describing a variety of structural cardiac malformations at birth. CHD affects approximately 1 % of live births, generating a large adult population with these abnormalities. Pulmonary hypertension (PH) in patients with adult CHD is heterogeneous, based on the type of defect and associated conditions, but is a known cause of adverse outcome.

**Method:**

We retrieved death certificate data from the CDC-WONDER database using ICD codes (I27.0, I27.2, I27.8, and I27.9., and Q20-26). Crude mortality rates (CMRs) and age-adjusted mortality rates (AAMRs) per 100,000 persons were calculated. Temporal trends were examined using the annual percent change (APC) and average annual percent change (AAPC) determined by Joinpoint regression**.**

**Result:**

From 1999 to 2020, an overall declining pattern was observed in the mortality rate. Men and women with congenital heart disease and pulmonary hypertension in the U.S. experienced a statistically significant decline in mortality rates. In terms of races, among White individuals, the decline was the most pronounced. All four U.S. census regions experienced statistically significant declines in mortality due to pulmonary hypertension among individuals with congenital heart disease. The rate of decline was steeper in rural areas compared to urban ones.

**Conclusion:**

This study highlights that previously implemented targeted interventions significantly contributed to the reduction of mortality amongst patiemnts with congenital heart disease and pulmonary hypertension in the U.S. Still improvements are required in certain areas, including female gender, Hispanic or Latino, and the Northeast and West regions.

## Graphical abstract

**Figure:**
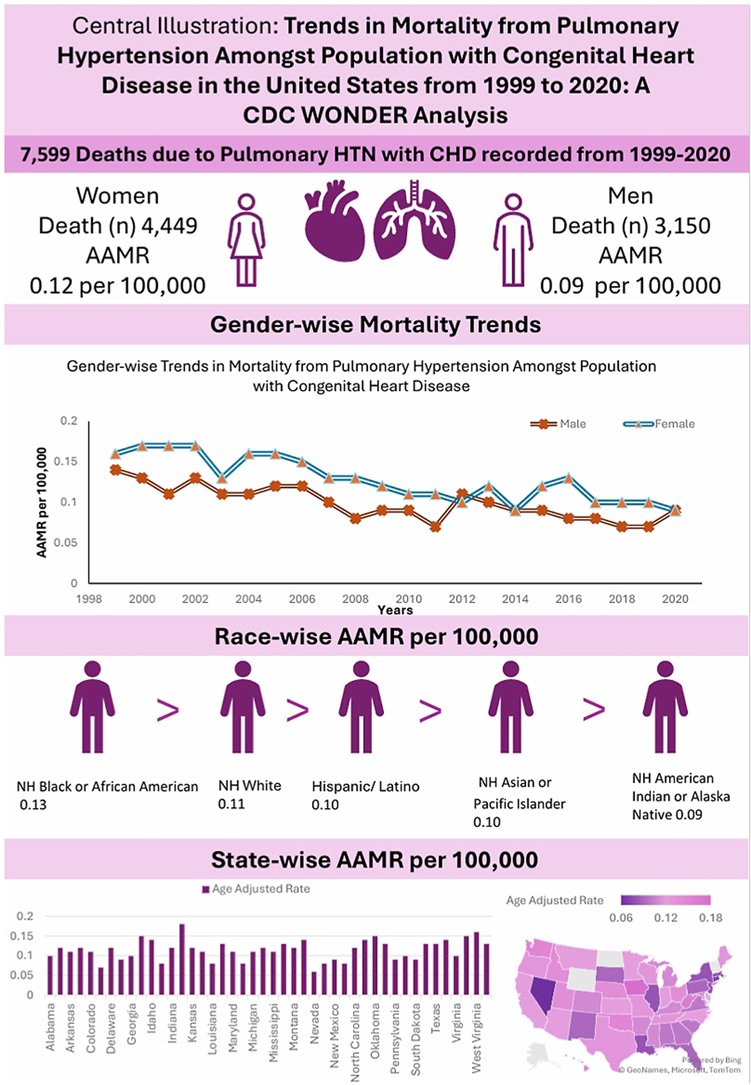

## 1 Introduction

Congenital heart disease (CHD) is a range of structural abnormalities of the heart present at birth [1]. It affects a significant number of individuals in the United States (US). It is estimated that approximately 1 % of live births are affected by CHD, leading to a substantial population of adults living with these conditions [2]. Pulmonary hypertension (PH) is a condition characterized by elevated blood pressure in the arteries that supply blood to the lungs. It is a known complication of various underlying conditions, including CHD, and can lead to significant morbidity, including exercise intolerance, heart failure, and reduced quality of life [3]. It is estimated that in 2024, 0.29 million Americans greater than 20 years were living with CHD [4]. In the U.S., the overall prevalence of CHD in the adult population is estimated to be around 0.8 % [5].

The prevalence of PH in adults with CHD varies depending on the specific type of defect and other factors, but it is a well-recognized contributor to adverse outcomes [6]. PH increases the all-cause mortality rate of adults with CHD more than 2-fold compared with patients without PH. Morbid complications including heart failure and arrhythmia occurs with a 3-fold higher risk compared with patients without PH [[7], [8], [9]].

Trends and disparities in CHD-related mortality and PH-related mortality among US adults have been discussed separately [10,11], but their association remains unexplored. It is crucial to understand the temporal trends of PH-related mortality in CHD patients to formulate targeted prevention strategies and improve patient care. This study utilizes the Centers for Disease Control and Prevention's (CDC) WONDER (Wide-ranging Online Data for Epidemiologic Research) database to examine national trends in PH-related mortality among patients with CHD in the United States from 1999 to 2020. By analyzing this comprehensive dataset, our study aims to identify patterns and shifts in mortality rates over time, assess potential demographic disparities, and explore the impact of evolving regional variations. The findings of this study will help future research aimed at reducing PH-related deaths, providing valuable insights for public health practitioners and policymakers, and improving the quality of life for individuals with congenital heart diseases.

## 2 Methods

This study is exempted from institutional review board (IRB) approval because of the usage of a deidentified government-issued public use database.

### 2.1 Data source

We obtained mortality data from the Centers for Disease Control and Prevention Wide-Ranging Online Data for Epidemiologic Research (CDC WONDER) Multiple Cause-of-Death Public Use database for the period January 1, 1999, through December 31, 2020. Death certificate records listing IE and renal failure among any of the underlying or contributing causes of death were identified via the International Statistical Classification of Diseases, Tenth Revision, Clinical Modification (ICD-10 codes). Specifically, we used codes I27.0 (Primary pulmonary hypertension), I27.2 (Other secondary pulmonary hypertension), I27.8 (Other specified pulmonary heart diseases), and I27.9 (Pulmonary heart disease, unspecified) for Pulmonary Hypertension (PH), and Q20 to Q26 for congenital heart disease (CHD), which have been validated and widely employed in prior epidemiological literature to accurately classify both these conditions, ensuring comparability with existing research [12,13]. The mortality data were collected for all age groups.

### 2.2 Data extraction

Information regarding deaths in patients with PH and CHD, population sizes, years, demographics such as age, gender, and ethnicity or racial background, along with census regions, urbanization classifications, states and place of death, was extracted. Race/ethnicity was coded according to U.S. Office of Management and Budget regulations and was used as stated on death certificates, with their classifications being as non-Hispanic (NH) White, NH Black, NH American Indian or Alaska Native, NH Asian or Pacific Islander, and Hispanic or Latino [14]. Moreover, regions were defined per U.S. Census Bureau classification as Northeast, Midwest, South, and the West [15]. The National Center for Health Statistics Urban-Rural Classification Scheme was used to categorize the populations, which were based on the 2013 U S. Census classification. It comprises the urban population that includes large metropolitan areas with a population of ≥1 million and medium/small metro areas with a population of 50,000–999,999, and the rural regions with a population of <50,000 [16]. The place of death categories included different medical settings like inpatient facilities, outpatient departments, emergency departments, dead on arrival, and unknown status individuals, as well as home, hospice, nursing home, or long-term care facilities.

### 2.3 Statistical analysis

To analyze national trends in patients with concomitant PH and CHD, we calculated crude mortality rates (CMRs) and age-adjusted mortality rates (AAMRs) per 100,000 population from 1999 to 2023, stratified by age groups, year, gender, race/ethnicity, census regions, states, and place of death. The CMRs and AAMRs per 100,000 population for urban-rural classification were computed from 1999 to 2020. The rates were calculated using 95 % confidence intervals (CIs). We determined CMRs by dividing the number of concomitant PH and CHD deaths by the corresponding population in the U.S. that year. At the same time, AAMRs were determined by standardizing the deaths to the U.S. population in 2000 [17]. To characterize temporal trends, we applied the Joinpoint Regression Program (Joinpoint V 4.9.0.0, National Cancer Institute) [17]. Using this tool, we computed the annual percent change (APC) in AAMR and calculated the 95 % confidence intervals (CIs). This method employs log-linear regression models to detect significant changes in AAMR over time when temporal fluctuations are present. The Weighted BIC test was utilized to derive APCs and their 95 % CIs for the selected line segments linking join points. Through 2-tailed t-testing, APCs were considered to be increasing or decreasing based on whether the slope indicating the change in mortality significantly differed from the null. A significant p-value was established at ≤0.05.

## 3 Results

### 3.1 Overall

Between 1999 and 2020, there was a statistically significant decline in mortality due to pulmonary hypertension among individuals with congenital heart disease in the U.S. According to the CDC WONDER data, the Annual Percent Change (APC) in mortality rates over the entire study period was −2.98 % (95 % CI: −3.52 % to −2.45 %). This indicates a consistent and notable decrease in mortality across the 21-year period. The negative APC suggests an average yearly reduction in death rates. The same APC value was observed for the Average Annual Percent Change (AAPC) across the full range of 1999–2020, indicating no joinpoints or changes in the trend's direction during the studied time period (Supplementary Table 1).

### 3.2 Sex stratification

**Fig. 1:**
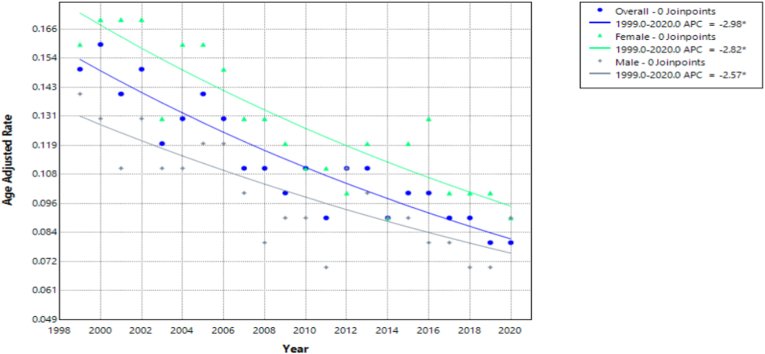
Age-adjusted mortality rates (AAMRs) per 100,000 individuals stratified by sex/gender in the United States, 1999 to 2020.

Over the study period, both females and males with congenital heart disease and pulmonary hypertension in the U.S. experienced a statistically significant decline in mortality rates, as indicated by the CDC WONDER data. For females, the Annual Percent Change (APC) in mortality was −2.82 % (95 % CI: −3.51 % to −2.12 %; p-value <0.000001). The Average Annual Percent Change (AAPC) for the full study period matched the APC value, indicating that the mortality decline was consistent without any significant shifts or reversals over time. Similarly, males also demonstrated a significant reduction in mortality. The APC was −2.57 % (95 % CI: −3.35 % to −1.80 %; p-value = 0.000001). The AAPC value mirrored this, further reinforcing the sustained nature of the decline across the entire study period (Fig. 1, Supplementary Table 2).

### 3.3 Race stratification

Mortality from pulmonary hypertension in individuals with congenital heart disease declined across all racial and ethnic groups, though the magnitude and certainty of these trends varied.

White individuals showed the most pronounced decline, with an APC of −3.19 % (95 % CI: −3.87 % to −2.49 %; p < 0.000001). The matching AAPC confirmed a steady, uninterrupted decrease over the 21-year period. Black or African American individuals also experienced a significant decline (APC: −2.71 %; 95 % CI: −3.75 % to −1.65 %; p = 0.000034), with a consistent AAPC.

**Fig. 2:**
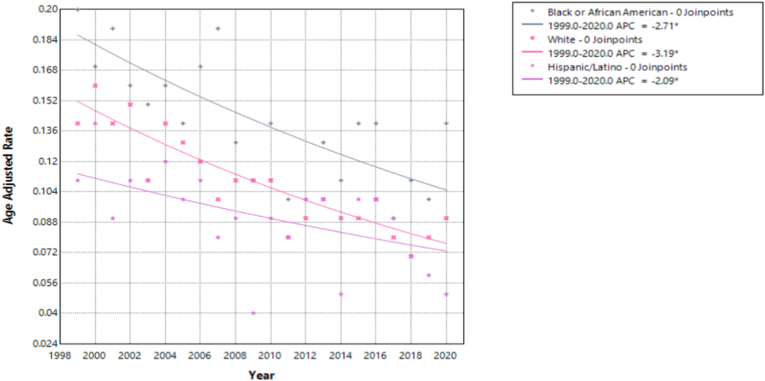
Age-adjusted mortality rates (AAMRs) per 100,000 individuals stratified by race/ethnicity in the United States, 1999 to 2020.

Hispanic/Latino individuals had a smaller, yet statistically significant, reduction (APC: −2.09 %; 95 % CI: −3.68 % to −0.48 %; p = 0.0139). While still meaningful, the wider confidence interval suggests greater variability and less certainty (Fig. 2, Supplementary Tables 3 and 4).

### 3.4 Census region

Each of the four U.S. census regions i.e. Northeast, Midwest, South and West, saw a statistically significant decrease in deaths caused by pulmonary hypertension in individuals with congenital heart disease over the period from 1999 to 2020, though the rate of decline differed among them.

The South (Census Region 3) demonstrated the most substantial annual reduction, with an Annual Percent Change (APC) of −3.34 % (95 % CI: −4.45 % to −2.21 %; p-value = 0.000005). The Average Annual Percent Change (AAPC) matched the APC, suggesting a consistent and strong downward trend throughout the study period.

The Midwest (Census Region 2) followed with an APC of −2.38 % (95 % CI: −3.13 % to −1.63 %; p-value = 0.000002), indicating a statistically significant decline. The AAPC was identical, reflecting a steady improvement over time.

**Fig. 3:**
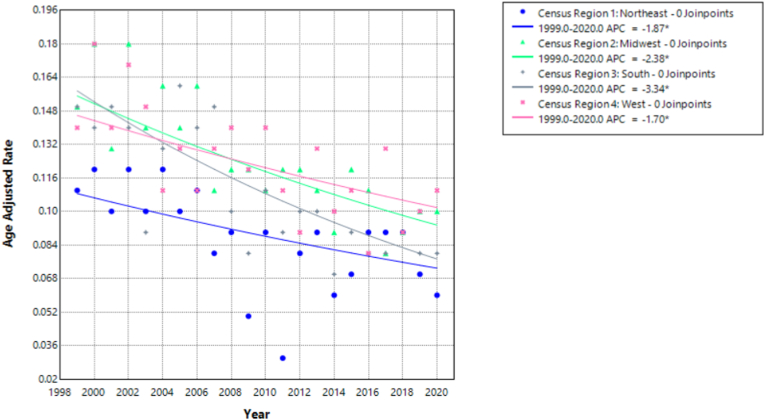
Age-adjusted mortality rates (AAMRs) per 100,000 individuals stratified by census region in the United States, 1999 to 2020.

Both the Northeast (Census Region 1) and the West (Census Region 4) exhibited the smallest rates of decline, with APCs of −1.87 % (95 % CI: −3.00 % to −0.74 %, p = 0.0027) and −1.70 % (95 % CI: −2.70 % to −0.69 %, p = 0.0023), respectively (Fig. 3, Supplementary Table 5).

### 3.5 Rural urban stratification

Statistically significant reductions in deaths from pulmonary hypertension among individuals with congenital heart disease were observed in both urban and rural U.S. populations between 1999 and 2020.

In rural populations, the Annual Percent Change (APC) was −3.31 % (95 % CI: −4.53 % to −2.07 %; p-value = 0.000022). This indicates a strong and statistically significant decrease in mortality over the study period. The Average Annual Percent Change (AAPC) mirrored the APC, suggesting a consistent and steady improvement in outcomes for rural residents.

**Fig. 4:**
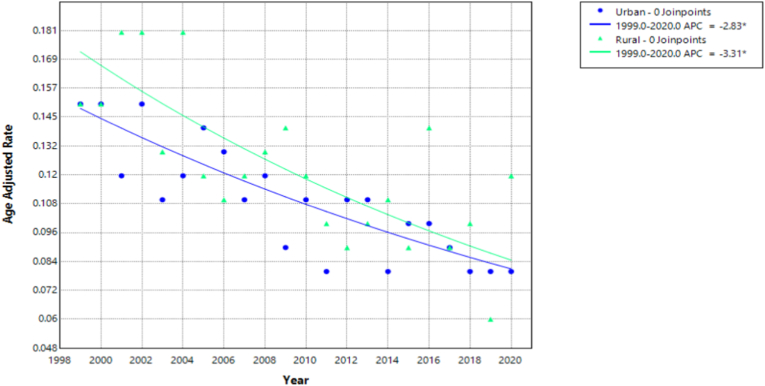
Age-adjusted mortality rates (AAMRs) per 100,000 individuals stratified by urbanization in the United States, 1999 to 2020.

Urban populations also saw a significant reduction in mortality, with an APC of −2.83 % (95 % CI: −3.59 % to −2.06 %; p-value <0.000001). While this decline is also significant, it is slightly less pronounced than in rural areas (Fig. 4, Supplementary Table 6).

### 3.6 Age stratification

Age-related trends in mortality due to pulmonary hypertension among individuals with congenital heart disease revealed a striking U-shaped pattern (high in infancy, lowest in childhood and adolescence, then rising steadily with age). Infants under 1 year were the most affected, accounting for 26.45 % of all deaths, with a crude mortality rate of 2.31 per 100,000 (95 % CI: 2.21–2.41) despite comprising a small portion of the population. In contrast, children aged 1–4 had a much lower rate of 0.18 (95 % CI: 0.17–0.19), contributing 8.28 % of deaths, while those aged 5–14 had the lowest burden, with just 282 deaths and a crude rate of 0.03 (95 % CI: 0.03–0.03). Mortality rates remained low through adolescence and early adulthood but gradually increased with age thereafter. Adults aged 25–34 had a rate of 0.06 (95 % CI: 0.05–0.06), rising to 0.13 (95 % CI: 0.12–0.14) in those aged 65–74 and peaking at 0.28 (95 % CI: 0.25–0.31) in individuals aged 85 and older. However, the elderly contributed fewer absolute deaths, likely due to survivorship bias and a smaller population base. Overall, the total crude mortality rate across all age groups was 0.11 per 100,000 (Supplementary Tables 7 and 8).

### 3.7 Place of death

The data on place of death for individuals with pulmonary hypertension associated with congenital heart disease showed that the majority of deaths, 5280 out of the total reported, occurred in inpatient medical facilities. A significant portion, 1150 deaths, occurred at home, indicating that some patients either preferred to stay at home during their final days or lacked access to timely medical care. Outpatient or emergency room settings accounted for 603 deaths. Nursing homes and long-term care facilities reported 250 deaths, showing that some patients lived with chronic, advanced forms of the disease in such settings. The number of deaths in hospice facilities was relatively low, at 101, which reflects an underutilization of hospice or palliative care services for this condition. Other locations, including those labeled as “Other” and “Unknown,” accounted for a small percentage of the deaths. Forty-nine individuals were reported dead on arrival, which may represent sudden, unexpected deaths. Eighteen deaths were listed as occurring in unknown locations, and data for deaths in facilities with unknown status was suppressed, likely due to privacy rules around small counts. Overall, the data indicates that while most patients with this condition die in clinical settings, there is a significant proportion who die outside of structured care (Supplementary Table 9).

### 3.8 State-wise stratification

**Fig. 5:**
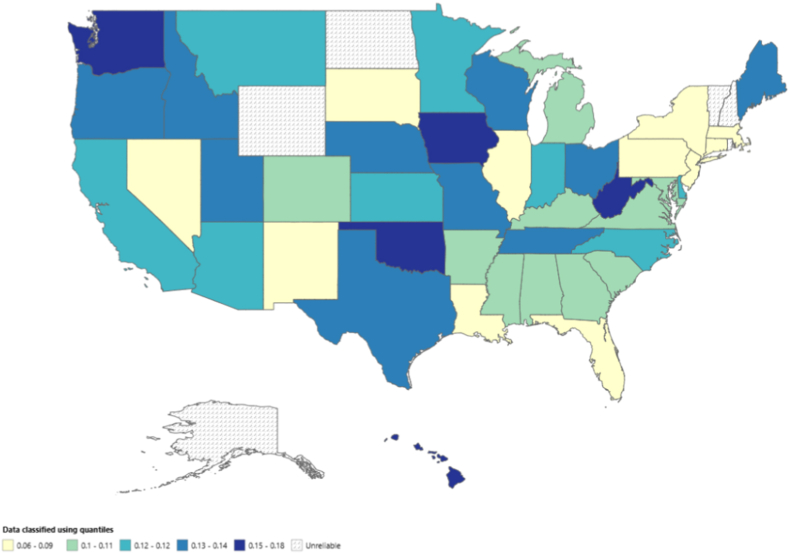
Age-adjusted mortality rates (AAMRs) per 100,000 individuals stratified by state in the United States, 1999 to 2020.

The mortality data for pulmonary hypertension among individuals with congenital heart disease across different U.S. states revealed notable variations in both the number of deaths and mortality rates. California reported the highest number of deaths, with 979 fatalities, accounting for 12.88 % of the total deaths in the dataset. Texas followed closely with 647 deaths (8.51 %) and Ohio with 407 deaths (5.36 %). Other states with high death counts included Florida (364 deaths, 4.79 %) and New York (350 deaths, 4.61 %). Age-adjusted mortality rates showed that Iowa had the highest rate at 0.17, followed by Ohio (0.16), West Virginia (0.16), Washington (0.15), and Utah (0.15). These states displayed a higher burden of mortality in relation to their populations, signaling a need for targeted healthcare interventions in these areas. In contrast, several states, such as Alaska, District of Columbia, North Dakota, New Hampshire, Rhode Island, and Vermont, had rates labeled as “Unreliable,” which can be attributed to smaller sample sizes or statistical limitations that affect the reliability of the data in these regions. On the other hand, some states reported the lowest age-adjusted mortality rates, with Nevada having the lowest at 0.06, followed by New Jersey and New York, both with a rate of 0.08 (Fig. 5, Supplementary Table 10).

### 3.9 Discussion

To the best of our knowledge, this is the first large-scale, population-based study to provide a modern look at the risk of death from pulmonary hypertension (PH) after being diagnosed with congenital heart disease (CHD). Our research shows that the risk of PH is not fixed; it changes based on several factors, including race, age, sex, how much time has passed since diagnosis, and the region where the diagnosis occurred. By analyzing data from 2000 to 2020, we identified significant patterns in mortality among people living with both PH and CHD. These patterns suggest a complex relationship between patient outcomes and the specific types of conditions they have, highlighting areas where targeted healthcare strategies could have the biggest impact. While overall death rates linked to CHD and PH have gone down over the past two decades, the decline has been slower for women. We also found clear racial disparities—Black individuals faced the highest mortality rates and saw the least improvement over time. Regionally, death rates were especially high in the Northeastern U.S. and in urban settings, compared to other areas.

Over the past 20 years, our analysis shows that overall mortality from pulmonary hypertension (PH) in individuals with congenital heart disease (CHD) has steadily declined. This positive trend is largely thanks to major advancements in medical care. As a result, people with adult congenital heart disease (ACHD) are now living significantly longer than in the past [18]. Breakthroughs in pediatric care have played a key role, leading to a growing number of adults living with CHD and related pulmonary hypertension (PH) [18]. Early interventions to repair heart defects, along with the timely use of advanced medical treatments, have not only improved quality of life but also appear to boost survival rates. Additionally, progress in diagnostic tools—like transthoracic echocardiography, cardiac catheterization, MRI, and CT scans—has greatly improved how clinicians manage CHD and PH, ultimately contributing to better outcomes for patients [[19], [20], [21], [22], [23], [24]]. Moreover, the Bosentan Randomized Trial of Endothelin Antagonist Therapy-5 (BREATHE-5) influenced revisions to the ACC/AHA guidelines significantly, which now endorse bosentan as a first-line treatment option for patients with PH-CHD [25]. Together all these interventions have drastically declined the mortality rates amongst the patient population.

In individuals with congenital heart disease (CHD), pulmonary hypertension (PH) often develops later on due to left-to-right shunt lesions or conditions that obstruct the left side of the heart, leading to postcapillary hypertension. Common congenital defects associated with this include ventricular septal defects (VSD), atrial septal defects (ASD), and a persistent ductus arteriosus. Studies have shown that the risk of developing pulmonary hypertension (PH) is closely linked to the size of the heart defect—larger defects typically carry a higher risk. Among individuals with CHD, the most common cause of PH is left heart disease, which is categorized under Group 2 in the clinical classification of pulmonary hypertension [26]. While the main reason for CHD-PH is the size and type of congenital heart defect, it looks like other things, like genetics, epigenetics, and environmental reasons, also contribute. Research shows that approximately 6 % of those affected with CHD-PH have mutations in the BMPR2 gene [27]. About 75 % of people with familial PH and 25 % of those with idiopathic PH have BMPR2 gene changes. Alongside BMPR2, changes in the ALK-1 gene, which leads to the formation of blood vessels and repairs, are also implicated in PH [28].

According to our analysis, females with CHD had a slightly increased AAMR for PH related deaths in comparison to males. This observation is consistent with findings from Chang et al., who also reported higher mortality rates in females compared to age-matched males, even though generally male sex has been recognized as a risk factor for mortality in PH-CHD cases [29]. Women with CHD appear to have a higher likelihood of developing pulmonary hypertension (PH). Data from the Dutch CONCOR registry, which includes adult CHD patients, showed that women were 35 % more likely than men to be diagnosed with PH [31]. In this cohort, atrial and ventricular septal defects were the most observed abnormalities and were more strongly associated with PH than other types of congenital defects, mirroring patterns seen in earlier research [30].

This increased vulnerability in women may be partly explained by hormonal influences and the physiological stress of pregnancy [31]. The significant cardiovascular changes during pregnancy—especially the shifts in blood volume and pressure—can exacerbate or even trigger pulmonary vascular disease, particularly in women whose pulmonary circulation is already compromised, potentially leading to poorer survival outcomes [31]. Moreover, women have smaller pulmonary arteries than men, and this may in part explain, along with other factors, why they might be more prone to developing PH [32,33].

Another possible explanation lies in the concept of the “estrogen paradox,” which aims to clarify the sex-based differences seen in PH [33]. While women are more prone to developing PH, they generally respond better to treatment and have higher survival rates once the disease is established. Animal studies have shown that estrogen can have both damaging and protective effects on the pulmonary vasculature. This dual role has led to growing interest in the scientific community, sparking detailed investigations into the role of estrogen in both experimental and clinical settings [34]. The present data on mortality differences between genders in relation to pulmonary hypertension (PH) and other forms of pulmonary hypertension (PH) in CHD remain limited thus more studies need to be conducted in this regard.

Our findings further highlight pronounced racial and ethnic disparities among individuals with PH associated with CHD. Throughout the study period, non-Hispanic Black individuals consistently exhibited the highest age-adjusted mortality rates (AAMRs). This pattern aligns with prior research, which has similarly reported increased mortality rates among Black populations.

Emerging evidence also suggests potential biological and clinical factors that may contribute to these disparities. For example, African American patients with pulmonary hypertension (PH) have been shown to respond less effectively to estrogen receptor antagonists compared to White patients [[35], [36], [37], [38]]. Another study reported that African Americans had significantly higher end-diastolic pulmonary regurgitation gradients than Caucasians and other racial groups, potentially indicating elevated mortality. Also, disorders such as CHD that promote the development of PH are noted more frequently in African American populations, possibly amplifying disease severity and influencing overall outcomes [[36], [37], [38]]. A study by Parikh et al. found that the higher mortality rates from PH in African Americans were somewhat mitigated when insurance status was considered [39]. The authors suggested that insurance status serves as a significant predictor of increased PH mortality. Further investigation into high-risk baseline characteristics, along with other social and economic determinants, could reveal opportunities for improving PH management [40].

In terms of age, the highest number of deaths occurred in infants under 1 year old. This may be attributed to the fact that in children, the symptoms of pulmonary hypertension (PH) are often mild or nonspecific, which can lead to delays in the diagnosis of pulmonary hypertension associated with congenital heart disease (PH-CHD) [41]. Beyond infancy, the highest mortality was observed in individuals aged 60 and above. However, thanks to advancements in medical technology and evidence-based guidelines, the overall mortality trend has shown a decline in adults between the ages of 18 and 60. This aligns with Khairy et al. study stating that the majority of related deaths are now beyond age 60 years, peaking at 80–84 years in CHD individuals [42]. Several previous studies have also implied that age increases the mortality risk in the CHD population. A notable multicenter retrospective study conducted across Europe, involving a substantial cohort of 1098 patients, identified several key predictors of mortality with advanced age being the most significant one [43].

We observed notable regional differences in age-adjusted mortality rates (AAMRs), with the highest rates reported in the Northeast. Furthermore, mortality rates were significantly higher in urban areas compared to rural regions. These disparities are largely influenced by regional differences in healthcare access, excessive burden on healthcare in rural areas, socioeconomic status, and lifestyle behaviors [44]. This contrasts with previous studies where the rural population faced higher mortalities. One possible explanation for this shift could be the increasing pressure on tertiary care hospitals, which may be stretched beyond their capacity, along with complications related to insurance coverage. Furthermore, the actual scope of these disparities may be even more significant than reported, as under-documentation is common in non-metropolitan areas, where hospitalization rates are lower and postmortem examinations are less frequently conducted [45,46].

### 3.10 Limitations

This study has several important limitations that should be considered when interpreting the findings. First, the primary data source, the CDC WONDER database, relies on death certificate records, which often lack detailed clinical information, limiting the depth of analysis.

Additionally, key determinants such as socioeconomic status, which play a crucial role in healthcare access and outcomes, were not assessed due to data limitations. The presence of unmeasured confounding variables may also have influenced mortality trends, introducing potential bias into the results. Finally, the absence of clinical data—such as biomarkers, treatment regimens, laboratory results, and specific therapeutic interventions—restricted our ability to fully explore the underlying contributors to the observed increase in PH -related mortality among CHD patients.

## 4 Conclusion

This study highlights significant disparities in mortality among individuals with pulmonary hypertension (PH) associated with congenital heart disease (CHD). While overall mortality has declined over the past two decades due to medical advancements, women and non-Hispanic Black individuals consistently experienced higher age-adjusted mortality rates. Infants under one year and adults over 60 had the highest mortality, with improved outcomes seen in adults aged 18–60. Regional differences were also noted, with higher mortality in the Northeast and urban areas. These findings underscore the need for targeted, equitable healthcare strategies to address persistent gaps in care.

## CRediT authorship contribution statement

**Allahdad Khan:** Writing – review & editing, Writing – original draft, Project administration, Methodology, Conceptualization. **Tehreem Asghar:** Writing – original draft, Investigation. **Kumail Mustafa Ali:** Writing – review & editing, Investigation, Formal analysis, Data curation. **Muhammad Saeed:** Writing – original draft, Software. **Saniya Ishtiaq:** Writing – original draft, Data curation. **Fatima Ashfaq:** Writing – original draft, Visualization. **Jamil Nasrallah:** Writing – original draft, Methodology. **Laiba Yumn:** Writing – original draft, Investigation. **Usman Ali Akbar:** Writing – review & editing, Validation, Conceptualization. **Peter Collins:** Writing – review & editing, Supervision, Project administration, Methodology. **Raheel Ahmed:** Writing – review & editing, Validation, Conceptualization.

## Ethics approval and consent to participate

Not Applicable.

## Consent for publication

Not Applicable.

## Availability of data and material

Data used to conduct analysis is given in the supplementary file. Data not provided will be provided upon reasonable request to corresponding author.

## Data availability statement

Data used for this study is available at https://wonder.cdc.gov/

## Funding

This research received no specific grant from any funding agency in the public, commercial, or not-for-profit sectors.

## Declaration of competing interest

The authors declare the following financial interests/personal relationships which may be considered as potential competing interests:Raheel Ahmed reports was provided by Imperial College London National Heart and Lung Institute. If there are other authors, they declare that they have no known competing financial interests or personal relationships that could have appeared to influence the work reported in this paper.

## References

[1] Meng X., Song M., Zhang K., Lu W., Li Y., Zhang C., Zhang Y. (2020). Congenital heart disease: types, pathophysiology, diagnosis, and treatment options. MedComm.

[2] Centers for Disease Control and Prevention (CDC) (2025). Congenital heart defects (CHDs). https://www.cdc.gov/heart-defects/about/index.html#:%7E:text=What%20it%20is,CHDs%20and%20%22Heart%20Heroes.%22.

[3] Oldroyd S.H., Manek G., Bhardwaj A. (2025 Jan). StatPearls.

[4] Suresh Kumar Vasupradha, Sable, Craig Nakiwala, Dorothy Kassebaum, Nicholas (2024). Abstract 4142897: the burden of adult congenital heart disease in the United States. Circulation. A4142897-A4142897. 150. Suppl_1.

[5] Roos-Hesselink Jolien W., Pelosi Chiara, Brida Margarita, De Backer Julie, Ernst Sabine, Werner Budts, Baumgartner Helmut, Oechslin Erwin, Tobler Daniel, Kovacs Adrienne H., Di Salvo Giovanni, Kluin Jolanda, Gatzoulis Michael A., Diller Gerhard P. (2024). Surveillance of adults with congenital heart disease: current guidelines and actual clinical practice. Int J Cardiol.

[6] D'Alto M., Diller G.P. (2014 Sep). Pulmonary hypertension in adults with congenital heart disease and Eisenmenger syndrome: current advanced management strategies. Heart.

[7] Lowe B., Therrien J., Ionescu-Ittu R. (2011 Jul). Diagnosis of pulmonary hypertension in the congenital heart disease adult population: impact on outcomes. JACC (J Am Coll Cardiol).

[8] GBD 2017 Congenital Heart Disease Collaborators (2020 Mar). Global, regional, and national burden of congenital heart disease, 1990-2017: a systematic analysis for the Global Burden of Disease Study 2017. Lancet Child Adolesc Health.

[9] Gong C.L., Zhao H., Wei Y., Tysinger B., Goldman D.P., Williams R.G. (2020 Oct). Lifetime burden of adult congenital heart disease in the USA using a microsimulation model. Pediatr Cardiol.

[10] Lopez K.N., Morris S.A., Sexson Tejtel S.K., Espaillat A., Salemi J.L. (2020 Sep 22). US mortality attributable to congenital heart disease across the lifespan from 1999 through 2017 exposes persistent racial/ethnic disparities. Circulation.

[11] Kang M., Hart C.M., Kempker J.A., Veeraraghavan S., Trammell A.W. (2022 Nov). Pulmonary hypertension mortality trends in United States 1999-2019. Ann Epidemiol.

[12] Kang M., Hart C.M., Kempker J.A., Veeraraghavan S., Trammell A.W. (2022). Pulmonary hypertension mortality trends in United States, 1999–2019. Ann Epidemiol.

[13] Lopez K.N., Morris S.A., Sexson Tejtel S.K., Espaillat A., Salemi J.L. (2020). US mortality attributable to congenital heart disease across the lifespan from 1999 through 2017 exposes persistent racial/ethnic disparities. Circulation.

[14] Multiple cause of death, 1999-2020 request. https://wonder.cdc.gov/mcd-icd10.html.

[15] Ingram D.D., Franco S.J. (2014). NCHS urban-rural classification scheme for counties. Vital Health Stat 2013.

[16] Aggarwal R., Chiu N., Loccoh E.C., Kazi D.S., Yeh R.W., Wadhera R.K. (2021 Mar 23). Rural-urban disparities: diabetes, hypertension, heart disease, and stroke mortality among black and white adults, 1999-2018. J Am Coll Cardiol.

[17] Anderson R.N., Rosenberg H.M. (1998). Age standardization of death rates: implementation of the year 2000 standard. Natl Vital Stat Rep.

[18] Fathallah M., Krasuski R.A. (2018 Sep-Oct). A multifaceted approach to pulmonary hypertension in adults with congenital heart disease. Prog Cardiovasc Dis.

[19] Bossone E., Ferrara F., Grunig E. (2015). Echocardiography in pulmonary hypertension. Curr Opin Cardiol.

[20] D'Alto M., Bossone E., Opotowsky A.R., Ghio S., Rudski L.G., Naeije R. (2018). Strengths and weaknesses of echocardiography for the diagnosis of pulmonary hypertension. Int J Cardiol.

[21] Bossone E., Ferrara F., Grünig E. (2015 Nov). Echocardiography in pulmonary hypertension. Curr Opin Cardiol.

[22] Kasprzak J.D., Huttin O., Wierzbowska-Drabik K., Selton-Suty C. (2018). Imaging the right heart-pulmonary circulation unit: the role of ultrasound. Heart Fail Clin.

[23] Kiely D.G., Levin D., Hassoun P., Ivy D.D., Jone P.N., Bwika J., Kawut S.M., Lordan J., Lungu A., Mazurek J. (2019). EXPRESS: statement on imaging and pulmonary hypertension from the Pulmonary Vascular Research Institute (PVRI). Pulm Circ.

[24] Bossone E., Chessa M., Butera G., Carbone G.L., Bodini B.D., Mazza E., Ballotta A. (2003 Apr). Echocardiographic assessment of overt or latent unexplained pulmonary hypertension. Can J Cardiol.

[25] Galie′ N., Beghetti M., Gatzoulis M.A. (2006). Bosentan therapy in patients with Eisenmenger syndrome: a multicenter, double-blind, randomized, placebocontrolled study. Circulation.

[26] Simonneau G., Galie N., Rubin L.J. (2004). Clinical classification of pulmonary hypertension. J Am Coll Cardiol.

[27] Beghetti M. (2006 Sep). Hypertension artérielle pulmonaire des cardiopathies congénitales [Pulmonary hypertension associated with congenital heart disease]. Rev Mal Respir.

[28] McLoughlin P., Hyvelin J.M., Howell K. (2005 Jan 27). Pulmonary hypertension. N Engl J Med.

[29] Chang W.T., Weng S.F., Hsu C.H. (2016). Prognostic factors in patients with pulmonary hypertension-A nationwide cohort study. J Am Heart Assoc.

[30] Somerville J. (1998). The Denolin lecture: the woman with congenital heart disease. Eur Heart J.

[31] Warnes C.A. (2008). Sex differences in congenital heart disease: should a woman be more like a man?. Circulation.

[32] Mercuro G., Bassareo P.P., Mariucci E., Deidda M., Zedda A.M., Bonvicini M. (2014). Sex differences in congenital heart defects and genetically induced arrhythmias. J Cardiovasc Med.

[33] Lahm T., Tuder R.M., Petrache I. (2014). Progress in solving the sex hormone paradox in pulmonary hypertension. Am J Physiol Lung Cell Mol Physiol.

[34] Docherty C.K., Harvey K.Y., Mair K.M., Griffin S., Denver N., MacLean M.R. (2018). The role of sex in the pathophysiology of pulmonary hypertension. Adv Exp Med Biol.

[35] Singh H., Agarwal L., Jani C., Bhatt P., Hartley A., Shalhoub J., Kurman J.S., Al Omari O., Ahmed A., Marshall D.C., Salciccioli J.D. (2023 Jun 30). Pulmonary hypertension associated mortality in the United States from 2003 to 2020: an observational analysis of time trends and disparities. J Thorac Dis.

[36] Austin E.D., Lahm T., West J., Tofovic S.P., Johansen A.K., Maclean M.R., Alzoubi A., Oka M. (2013 Apr). Gender, sex hormones and pulmonary hypertension. Pulm Circ.

[37] Khush K.K., Shah S.J., Ristow B. (2007). Association of African American race with elevated pulmonary artery diastolic pressure: data from the Heart and Soul Study. J Am Soc Echocardiogr.

[38] Beall A.D., Nietert P.J., Taylor M.H. (2007). Ethnic disparities among patients with pulmonary hypertension associated with systemic sclerosis. J Rheumatol.

[39] Parikh K.S., Stackhouse K.A., Hart S.A. (2017). Health insurance and racial disparities in pulmonary hypertension outcomes. Am J Manag Care.

[40] Fiscella K., Franks P., Gold M.R. (2000). Inequality in quality: addressing socioeconomic, racial, and ethnic disparities in health care. JAMA.

[41] Berger R.M., Beghetti M., Humpl T., Raskob G.E., Ivy D.D., Jing Z.C., Bonnet D., Schulze-Neick I., Barst R.J. (2012). Clinical features of paediatric pulmonary hypertension: a registry study. Lancet.

[42] Khairy P., Ionescu‐Ittu R., Mackie A.S., Abrahamowicz M., Pilote L., Marelli A.J. (2010). Changing mortality in congenital heart disease. J Am Coll Cardiol.

[43] Kempny A., Hjortshøj C.S., Gu H., Li W., Opotowsky A.R., Landzberg M.J., Jensen A.S., Søndergaard L., Estensen M.‐E., Thilén U. (2017). Predictors of death in contemporary adult patients with Eisenmenger syndrome: a multicenter study. Circulation.

[44] Sekkarie A., Woodruff R.C., Casper M., Paul A.T., Vaughan A.S. (2025 Jan). Rural-urban disparities in cardiovascular disease mortality vary by poverty level and region. J Rural Health.

[45] Nuako A., Liu J., Pham G., Smock N., James A., Baker T. (2022 Feb 1). Quantifying rural disparity in healthcare utilization in the United States: analysis of a large midwestern healthcare system. PLoS One [Internet].

[46] Greenwood-Ericksen M.B., Kocher K. (2019 Apr 5). Trends in emergency department use by rural and urban populations in the United States. JAMA Netw Open.

