# Supplementary material for: Trends in mortality from pulmonary hypertension amongst population with congenital heart disease in the United States from 1999 to 2020: A CDC WONDER analysis
Source: Int J Cardiol Congenit Heart Dis. 2025 Jun 21;21:100602. doi: 10.1016/j.ijcchd.2025.100602 (PMC12274916; doi:10.1016/j.ijcchd.2025.100602)
Supplement: Multimedia component 1 [file mmc1.docx]

**Supplemental Table 1. Overall and Sex- Pulmonary hypertension related Mortality Amongst Population with Congenital Heart Disease in the United States from 1999 to 2020**

| **Year** | **Male** | **Female** | **Overall** |
| --- | --- | --- | --- |
| 1999 | 180 | 238 | 418 |
| 2000 | 161 | 226 | 387 |
| 2001 | 149 | 246 | 395 |
| 2002 | 172 | 250 | 422 |
| 2003 | 139 | 208 | 347 |
| 2004 | 157 | 242 | 399 |
| 2005 | 164 | 223 | 387 |
| 2006 | 146 | 222 | 368 |
| 2007 | 152 | 201 | 353 |
| 2008 | 134 | 208 | 342 |
| 2009 | 134 | 179 | 313 |
| 2010 | 140 | 206 | 346 |
| 2011 | 119 | 179 | 298 |
| 2012 | 145 | 171 | 316 |
| 2013 | 141 | 189 | 330 |
| 2014 | 124 | 159 | 283 |
| 2015 | 144 | 194 | 338 |
| 2016 | 135 | 191 | 326 |
| 2017 | 130 | 172 | 302 |
| 2018 | 124 | 173 | 297 |
| 2019 | 114 | 193 | 307 |
| 2020 | 146 | 179 | 325 |
| **Total** | **3,150** | **4,449** | **7,599** |

**Supplemental Table 2. Trends in mortality from pulmonary hypertension amongst population with congenital heart disease in the United States from 1999 to 2020 stratified by AAMR and sex**

| **Year** | **Men AAMR (95% CI)** | **Women AAMR (95% CI)** | **Overall AAMR (95% CI)** |
| --- | --- | --- | --- |
| 1999 | 0.14 (0.12–0.17) | 0.16 (0.14–0.18) | 0.15 (0.14–0.17) |
| 2000 | 0.13 (0.11–0.15) | 0.17 (0.15–0.19) | 0.16 (0.14–0.17) |
| 2001 | 0.11 (0.09–0.13) | 0.17 (0.15–0.20) | 0.14 (0.13–0.16) |
| 2002 | 0.13 (0.11–0.15) | 0.17 (0.15–0.19) | 0.15 (0.14–0.17) |
| 2003 | 0.11 (0.09–0.13) | 0.13 (0.11–0.15) | 0.12 (0.11–0.13) |
| 2004 | 0.11 (0.09–0.13) | 0.16 (0.14–0.18) | 0.13 (0.12–0.15) |
| 2005 | 0.12 (0.10–0.13) | 0.16 (0.14–0.18) | 0.14 (0.12–0.15) |
| 2006 | 0.12 (0.10–0.14) | 0.15 (0.13–0.17) | 0.13 (0.12–0.15) |
| 2007 | 0.10 (0.09–0.12) | 0.13 (0.11–0.15) | 0.11 (0.10–0.12) |
| 2008 | 0.08 (0.06–0.09) | 0.13 (0.11–0.15) | 0.11 (0.10–0.13) |
| 2009 | 0.09 (0.07–0.11) | 0.12 (0.10–0.14) | 0.10 (0.09–0.11) |
| 2010 | 0.09 (0.07–0.11) | 0.11 (0.10–0.13) | 0.11 (0.10–0.13) |
| 2011 | 0.07 (0.06–0.09) | 0.11 (0.09–0.13) | 0.09 (0.08–0.10) |
| 2012 | 0.11 (0.09–0.12) | 0.10 (0.08–0.12) | 0.11 (0.10–0.12) |
| 2013 | 0.10 (0.08–0.12) | 0.12 (0.10–0.14) | 0.11 (0.10–0.12) |
| 2014 | 0.09 (0.07–0.11) | 0.09 (0.07–0.10) | 0.09 (0.08–0.10) |
| 2015 | 0.09 (0.08–0.11) | 0.12 (0.10–0.14) | 0.10 (0.09–0.11) |
| 2016 | 0.08 (0.06–0.09) | 0.13 (0.11–0.14) | 0.10 (0.09–0.12) |
| 2017 | 0.08 (0.06–0.10) | 0.10 (0.09–0.12) | 0.09 (0.08–0.11) |
| 2018 | 0.07 (0.05–0.08) | 0.10 (0.08–0.12) | 0.09 (0.07–0.10) |
| 2019 | 0.07 (0.05–0.08) | 0.10 (0.09–0.12) | 0.08 (0.07–0.09) |
| 2020 | 0.09 (0.08–0.11) | 0.09 (0.08–0.11) | 0.08 (0.07–0.09) |
| **Total** | **0.09 (0.09–0.10)** | **0.12 (0.12–0.13)** | **0.12 (0.11–0.12)** |

**Supplemental Table 3. Trends in mortality from pulmonary hypertension amongst population with congenital heart disease in the United States from 1999 to 2020 stratified by race and ethnicity**

| **Year** | **NH White** | **NH Black or African American** | **NH Asian or Pacific Islander** | **Hispanic** | **Overall Population** |
| --- | --- | --- | --- | --- | --- |
| 1999 | 279 | 76 | Suppressed | 50 | 279,040,168 |
| 2000 | 254 | 62 | 14 | 52 | 281,421,906 |
| 2001 | 276 | 66 | 13 | 36 | 284,968,955 |
| 2002 | 298 | 60 | 15 | 44 | 287,625,193 |
| 2003 | 231 | 50 | 17 | 44 | 290,107,933 |
| 2004 | 257 | 66 | 15 | 56 | 292,805,298 |
| 2005 | 255 | 56 | 16 | 57 | 295,516,599 |
| 2006 | 234 | 60 | 14 | 58 | 298,379,912 |
| 2007 | 215 | 69 | 19 | 48 | 301,231,207 |
| 2008 | 218 | 51 | 16 | 54 | 304,093,966 |
| 2009 | 199 | 52 | 18 | 38 | 306,771,529 |
| 2010 | 217 | 53 | 23 | 48 | 308,745,538 |
| 2011 | 190 | 48 | 12 | 48 | 311,591,917 |
| 2012 | 208 | 37 | 15 | 54 | 313,914,040 |
| 2013 | 199 | 51 | 23 | 54 | 316,128,839 |
| 2014 | 185 | 48 | Suppressed | 41 | 318,857,056 |
| 2015 | 204 | 61 | 12 | 56 | 321,418,820 |
| 2016 | 195 | 64 | 10 | 54 | 323,127,513 |
| 2017 | 174 | 44 | 20 | 60 | 325,719,178 |
| 2018 | 175 | 48 | 18 | 52 | 327,167,434 |
| 2019 | 189 | 52 | 16 | 48 | 328,239,523 |
| 2020 | 206 | 56 | 18 | 43 | 329,484,123 |
| **Total** | **4858** | **1230** | **339** | **1066** | **6,746,356,647** |

### **Supplemental Table 4. Trends in Mortality from Pulmonary Hypertension Amongst Population with Congenital Heart Disease in the United States from 1999 to 2020 stratified by Race and Age-Adjusted Mortality Rate (AAMR)**

| **Year** | **NH White AAMR (95% CI)** | **NH Black or African American AAMR (95% CI)** | **NH Asian or Pacific Islander AAMR (95% CI)** | **Hispanic AAMR (95% CI)** |
| --- | --- | --- | --- | --- |
| 1999 | 0.14 (0.12–0.16) | 0.20 (0.16–0.25) | Suppressed | 0.11 (0.08–0.16) |
| 2000 | 0.16 (0.14–0.18) | 0.17 (0.13–0.23) | Unreliable | 0.14 (0.09–0.20) |
| 2001 | 0.14 (0.12–0.15) | 0.19 (0.14–0.24) | Unreliable | 0.09 (0.05–0.13) |
| 2002 | 0.15 (0.13–0.17) | 0.16 (0.12–0.21) | Unreliable | 0.11 (0.07–0.15) |
| 2003 | 0.11 (0.10–0.13) | 0.15 (0.11–0.20) | Unreliable | 0.11 (0.08–0.16) |
| 2004 | 0.14 (0.12–0.16) | 0.16 (0.12–0.21) | Unreliable | 0.12 (0.08–0.16) |
| 2005 | 0.13 (0.11–0.14) | 0.14 (0.10–0.18) | Unreliable | 0.10 (0.07–0.14) |
| 2006 | 0.12 (0.10–0.14) | 0.17 (0.13–0.22) | Unreliable | 0.11 (0.08–0.16) |
| 2007 | 0.10 (0.09–0.12) | 0.19 (0.15–0.24) | Unreliable | 0.08 (0.06–0.11) |
| 2008 | 0.11 (0.09–0.12) | 0.13 (0.09–0.17) | Unreliable | 0.09 (0.06–0.13) |
| 2009 | 0.11 (0.09–0.12) | 0.11 (0.08–0.14) | Unreliable | 0.04 (0.02–0.07) |
| 2010 | 0.11 (0.10–0.13) | 0.14 (0.10–0.18) | 0.13 (0.08–0.20) | 0.09 (0.06–0.12) |
| 2011 | 0.08 (0.07–0.10) | 0.10 (0.07–0.13) | Unreliable | 0.08 (0.06–0.12) |
| 2012 | 0.09 (0.08–0.11) | 0.10 (0.06–0.14) | Unreliable | 0.10 (0.07–0.14) |
| 2013 | 0.10 (0.08–0.11) | 0.13 (0.09–0.17) | 0.14 (0.08–0.21) | 0.10 (0.07–0.14) |
| 2014 | 0.09 (0.07–0.10) | 0.11 (0.08–0.15) | Suppressed | 0.05 (0.03–0.08) |
| 2015 | 0.09 (0.07–0.10) | 0.14 (0.10–0.18) | Unreliable | 0.10 (0.07–0.14) |
| 2016 | 0.10 (0.08–0.11) | 0.14 (0.11–0.18) | Unreliable | 0.10 (0.07–0.13) |
| 2017 | 0.08 (0.07–0.09) | 0.09 (0.06–0.12) | 0.11 (0.07–0.17) | 0.09 (0.07–0.13) |
| 2018 | 0.07 (0.06–0.08) | 0.11 (0.08–0.15) | Unreliable | 0.07 (0.05–0.10) |
| 2019 | 0.08 (0.07–0.09) | 0.10 (0.07–0.13) | Unreliable | 0.06 (0.04–0.09) |
| 2020 | 0.09 (0.07–0.10) | 0.14 (0.10–0.19) | Unreliable | 0.05 (0.04–0.08) |
| **Total** | **0.11 (0.11–0.11)** | **0.13 (0.12–0.14)** | **0.10 (0.09–0.11)** | **0.10 (0.09–0.10)** |

### **Supplemental Table 5. Trends in Mortality from Pulmonary Hypertension Amongst Population with Congenital Heart Disease in the United States from 1999 to 2020 stratified by census region**

| **Year** | **Northeast (AAMR) (Lower - Upper)** | **Midwest (AAMR) (Lower - Upper)** | **South (AAMR) (Lower - Upper)** | **West (AAMR) (Lower - Upper)** |
| --- | --- | --- | --- | --- |
| 1999 | 0.11 (0.08 - 0.14) | 0.15 (0.12 - 0.17) | 0.15 (0.13 - 0.18) | 0.14 (0.12 - 0.18) |
| 2000 | 0.12 (0.08 - 0.15) | 0.18 (0.15 - 0.22) | 0.14 (0.11 - 0.16) | 0.18 (0.14 - 0.22) |
| 2001 | 0.10 (0.08 - 0.13) | 0.13 (0.10 - 0.16) | 0.15 (0.12 - 0.17) | 0.14 (0.11 - 0.18) |
| 2002 | 0.12 (0.09 - 0.16) | 0.18 (0.14 - 0.21) | 0.14 (0.12 - 0.17) | 0.17 (0.14 - 0.21) |
| 2003 | 0.10 (0.08 - 0.14) | 0.14 (0.11 - 0.17) | 0.09 (0.07 - 0.11) | 0.15 (0.12 - 0.18) |
| 2004 | 0.12 (0.09 - 0.16) | 0.16 (0.13 - 0.19) | 0.13 (0.11 - 0.15) | 0.11 (0.08 - 0.14) |
| 2005 | 0.10 (0.07 - 0.13) | 0.14 (0.11 - 0.18) | 0.16 (0.14 - 0.19) | 0.13 (0.10 - 0.16) |
| 2006 | 0.11 (0.08 - 0.15) | 0.16 (0.13 - 0.20) | 0.14 (0.11 - 0.16) | 0.11 (0.08 - 0.13) |
| 2007 | 0.08 (0.06 - 0.11) | 0.11 (0.08 - 0.14) | 0.15 (0.13 - 0.18) | 0.13 (0.10 - 0.16) |
| 2008 | 0.09 (0.06 - 0.12) | 0.12 (0.10 - 0.16) | 0.10 (0.08 - 0.12) | 0.14 (0.11 - 0.17) |
| 2009 | 0.05 (0.03 - 0.08) | 0.12 (0.10 - 0.15) | 0.08 (0.07 - 0.10) | 0.12 (0.09 - 0.15) |
| 2010 | 0.09 (0.07 - 0.12) | 0.11 (0.08 - 0.13) | 0.11 (0.09 - 0.13) | 0.14 (0.11 - 0.17) |
| 2011 | 0.03 (0.02 - 0.05) | 0.12 (0.10 - 0.16) | 0.09 (0.07 - 0.11) | 0.11 (0.08 - 0.13) |
| 2012 | 0.08 (0.06 - 0.12) | 0.12 (0.10 - 0.16) | 0.10 (0.08 - 0.12) | 0.09 (0.07 - 0.12) |
| 2013 | 0.09 (0.06 - 0.12) | 0.11 (0.09 - 0.14) | 0.10 (0.08 - 0.12) | 0.13 (0.10 - 0.16) |
| 2014 | 0.06 (0.04 - 0.09) | 0.09 (0.06 - 0.11) | 0.07 (0.05 - 0.08) | 0.10 (0.07 - 0.12) |
| 2015 | 0.07 (0.05 - 0.10) | 0.12 (0.10 - 0.15) | 0.09 (0.07 - 0.11) | 0.11 (0.09 - 0.14) |
| 2016 | 0.09 (0.07 - 0.13) | 0.11 (0.09 - 0.14) | 0.08 (0.06 - 0.09) | 0.08 (0.06 - 0.11) |
| 2017 | 0.09 (0.06 - 0.12) | 0.08 (0.06 - 0.11) | 0.08 (0.06 - 0.10) | 0.13 (0.10 - 0.16) |
| 2018 | 0.09 (0.06 - 0.12) | 0.09 (0.07 - 0.12) | 0.09 (0.07 - 0.11) | 0.09 (0.07 - 0.12) |
| 2019 | 0.07 (0.04 - 0.09) | 0.10 (0.07 - 0.13) | 0.08 (0.06 - 0.10) | 0.10 (0.07 - 0.12) |
| 2020 | 0.06 (0.04 - 0.08) | 0.10 (0.08 - 0.13) | 0.08 (0.06 - 0.10) | 0.11 (0.09 - 0.14) |
| **Total** | 0.09 (0.09 - 0.10) | 0.12 (0.12 - 0.13) | 0.12 (0.11 - 0.12) | 0.12 (0.12 - 0.13) |

### **Supplemental Table 6: Trends in Mortality from Pulmonary Hypertension Amongst Population with Congenital Heart Disease in the United States from 1999 to 2020 stratified by urban and rural**

| Year | Urban (AAMR) (Lower - Upper) | Rural (AAMR) (Lower - Upper) |
| --- | --- | --- |
| 1999 | 0.15 (0.14 - 0.17) | 0.15 (0.11 - 0.20) |
| 2000 | 0.15 (0.13 - 0.17) | 0.15 (0.12 - 0.20) |
| 2001 | 0.12 (0.11 - 0.14) | 0.18 (0.15 - 0.23) |
| 2002 | 0.15 (0.14 - 0.17) | 0.18 (0.14 - 0.22) |
| 2003 | 0.11 (0.10 - 0.13) | 0.13 (0.10 - 0.17) |
| 2004 | 0.12 (0.11 - 0.13) | 0.18 (0.14 - 0.23) |
| 2005 | 0.14 (0.12 - 0.16) | 0.12 (0.09 - 0.16) |
| 2006 | 0.13 (0.12 - 0.15) | 0.11 (0.08 - 0.14) |
| 2007 | 0.11 (0.09 - 0.12) | 0.12 (0.09 - 0.16) |
| 2008 | 0.12 (0.10 - 0.13) | 0.13 (0.10 - 0.17) |
| 2009 | 0.09 (0.08 - 0.11) | 0.14 (0.10 - 0.18) |
| 2010 | 0.11 (0.10 - 0.12) | 0.12 (0.09 - 0.16) |
| 2011 | 0.08 (0.07 - 0.09) | 0.10 (0.08 - 0.14) |
| 2012 | 0.11 (0.10 - 0.12) | 0.09 (0.06 - 0.12) |
| 2013 | 0.11 (0.09 - 0.12) | 0.10 (0.07 - 0.13) |
| 2014 | 0.08 (0.07 - 0.10) | 0.11 (0.08 - 0.15) |
| 2015 | 0.10 (0.09 - 0.11) | 0.09 (0.07 - 0.13) |
| 2016 | 0.10 (0.08 - 0.11) | 0.14 (0.10 - 0.18) |
| 2017 | 0.09 (0.08 - 0.10) | 0.09 (0.06 - 0.12) |
| 2018 | 0.08 (0.07 - 0.10) | 0.10 (0.07 - 0.14) |
| 2019 | 0.08 (0.07 - 0.09) | 0.06 (0.04 - 0.09) |
| 2020 | 0.08 (0.07 - 0.09) | 0.12 (0.09 - 0.16) |
| Total | 0.12 (0.11 - 0.12) | 0.13 (0.12 - 0.14) |

### **Supplemental Table 7: Trends in Mortality from Pulmonary Hypertension Amongst Population with Congenital Heart Disease in the United States from 1999 to 2020 stratified by age**

| **Age group** | **Deaths** |
| --- | --- |
| < 1 year | 86913756 |
| 1-4 years | 629 |
| 5-14 years | 282 |
| 15-24 years | 404 |
| 25-34 years | 516 |
| 35-44 years | 673 |
| 45-54 years | 785 |
| 55-64 years | 742 |
| 65-74 years | 643 |
| 75-84 years | 586 |
| 85+ years | 329 |

### **Supplemental Table 8: Trends in Mortality from Pulmonary Hypertension Amongst Population with Congenital Heart Disease in the United States from 1999 to 2020 stratified by age** **in the United States from 1999 to 2020**

| **Age Group** | **Crude Rate (LL - UL)** |
| --- | --- |
| < 1 year | 2.31 (2.21 - 2.41) |
| 1-4 years | 0.18 (0.17 - 0.19) |
| 5-14 years | 0.03 (0.03 - 0.03) |
| 15-24 years | 0.04 (0.04 - 0.05) |
| 25-34 years | 0.06 (0.05 - 0.06) |
| 35-44 years | 0.07 (0.07 - 0.08) |
| 45-54 years | 0.08 (0.08 - 0.09) |
| 55-64 years | 0.10 (0.09 - 0.10) |
| 65-74 years | 0.13 (0.12 - 0.14) |
| 75-84 years | 0.20 (0.18 - 0.21) |
| 85+ years | 0.28 (0.25 - 0.31) |

### **Supplemental Table 9: Trends in Mortality from Pulmonary Hypertension Amongst Population with Congenital Heart Disease in the United States from 1999 to 2020 stratified by place of death**

| **Place of Death** | **Deaths** |
| --- | --- |
| Medical Facility - Inpatient | 5280 |
| Medical Facility - Outpatient or ER | 603 |
| Medical Facility - Dead on Arrival | 49 |
| Medical Facility - Status unknown | Suppressed |
| Decedent's home | 1150 |
| Hospice facility | 101 |
| Nursing home/long term care | 250 |
| Other | 144 |
| Place of death unknown | 18 |

### **Supplemental Table 10: Trends in Mortality from Pulmonary Hypertension Amongst Population with Congenital Heart Disease in the United States from 1999 to 2020 stratified by states and Age-Adjusted Mortality Rate (AAMR)**

| **State** | **AAMR (LL - UL)** |
| --- | --- |
| Alabama | 0.10 (0.08 - 0.12) |
| Alaska | Unreliable (0.04 - 0.15) |
| Arizona | 0.12 (0.10 - 0.14) |
| Arkansas | 0.11 (0.08 - 0.14) |
| California | 0.12 (0.11 - 0.12) |
| Colorado | 0.11 (0.09 - 0.14) |
| Connecticut | 0.07 (0.05 - 0.10) |
| Delaware | 0.12 (0.07 - 0.18) |
| District of Columbia | Unreliable (0.08 - 0.21) |
| Florida | 0.09 (0.08 - 0.10) |
| Georgia | 0.10 (0.08 - 0.11) |
| Hawaii | 0.15 (0.10 - 0.20) |
| Idaho | 0.14 (0.10 - 0.19) |
| Illinois | 0.08 (0.07 - 0.09) |
| Indiana | 0.12 (0.11 - 0.14) |
| Iowa | 0.18 (0.15 - 0.22) |
| Kansas | 0.12 (0.09 - 0.15) |
| Kentucky | 0.11 (0.09 - 0.14) |
| Louisiana | 0.08 (0.06 - 0.10) |
| Maine | 0.13 (0.09 - 0.19) |
| Maryland | 0.11 (0.09 - 0.13) |
| Massachusetts | 0.08 (0.06 - 0.09) |
| Michigan | 0.11 (0.09 - 0.12) |
| Minnesota | 0.12 (0.10 - 0.14) |
| Mississippi | 0.11 (0.09 - 0.14) |
| Missouri | 0.13 (0.11 - 0.15) |
| Montana | 0.12 (0.07 - 0.18) |
| Nebraska | 0.14 (0.11 - 0.18) |
| Nevada | 0.06 (0.04 - 0.09) |
| New Hampshire | Unreliable (0.04 - 0.10) |
| New Jersey | 0.08 (0.07 - 0.09) |
| New Mexico | 0.09 (0.06 - 0.13) |
| New York | 0.08 (0.07 - 0.09) |
| North Carolina | 0.12 (0.10 - 0.14) |
| North Dakota | Unreliable (0.05 - 0.18) |
| Ohio | 0.14 (0.12 - 0.15) |
| Oklahoma | 0.15 (0.12 - 0.18) |
| Oregon | 0.13 (0.10 - 0.15) |
| Pennsylvania | 0.09 (0.08 - 0.10) |
| Rhode Island | Unreliable (0.04 - 0.11) |
| South Carolina | 0.10 (0.08 - 0.12) |
| South Dakota | 0.09 (0.05 - 0.14) |
| Tennessee | 0.13 (0.11 - 0.15) |
| Texas | 0.13 (0.12 - 0.14) |
| Utah | 0.14 (0.11 - 0.18) |
| Vermont | Unreliable (0.07 - 0.21) |
| Virginia | 0.10 (0.08 - 0.11) |
| Washington | 0.15 (0.13 - 0.17) |
| West Virginia | 0.16 (0.12 - 0.21) |
| Wisconsin | 0.13 (0.11 - 0.15) |
| Wyoming | Unreliable (0.09 - 0.25) |
